# Integrated programs for women with substance use issues and their children: a qualitative meta-synthesis of processes and outcomes

**DOI:** 10.1186/1477-7517-6-32

**Published:** 2009-11-20

**Authors:** Wendy Sword, Susan Jack, Alison Niccols, Karen Milligan, Joanna Henderson, Lehana Thabane

**Affiliations:** 1 School of Nursing, McMaster University, Hamilton, Ontario, Canada; 2 Department of Psychiatry and Behavioural Neurosciences, McMaster University, Hamilton, Ontario, Canada; 3 Psychology and Research, Integra, Toronto, Ontario, Canada; 4 Child, Youth & Family Program, Centre for Addiction and Mental Health, Toronto, Ontario, Canada; 5 Department of Clinical Epidemiology and Biostatistics, McMaster University, Hamilton, Ontario, Canada

## Abstract

**Background:**

There is a need for services that effectively and comprehensively address the complex needs of women with substance use issues and their children. A growing body of literature supports the relevance of integrated treatment programs that offer a wide range of services in centralized settings. Quantitative studies suggest that these programs are associated with positive outcomes. A qualitative meta-synthesis was conducted to provide insight into the processes that contribute to recovery in integrated programs and women's perceptions of benefits for themselves and their children.

**Methods:**

A comprehensive search of published and unpublished literature to August 2009 was carried out for narrative reports of women's experiences and perceptions of integrated treatment programs. Eligibility for inclusion in the meta-synthesis was determined using defined criteria. Quality assessment was then conducted. Qualitative data and interpretations were extracted from studies of adequate quality, and were synthesized using a systematic and iterative process to create themes and overarching concepts.

**Results:**

A total of 15 documents were included in the meta-synthesis. Women experienced a number of psychosocial processes during treatment that played a role in their recovery and contributed to favourable outcomes. These included: development of a sense of self; development of personal agency; giving and receiving of social support; engagement with program staff; self-disclosure of challenges, feelings, and past experiences; recognizing patterns of destructive behaviour; and goal setting. A final process, the motivating presence of children, sustained women in their recovery journeys. Perceived outcomes included benefits for maternal and child well-being, and enhanced parenting capacity.

**Conclusion:**

A number of distinct but interconnected processes emerged as being important to women's addiction recovery. Women experienced individual growth and transformative learning that led to a higher quality of life and improved interactions with their children. The findings support the need for programs to adopt practices that focus on improving maternal health and social functioning in an environment characterized by empowerment, safety, and connections. Women's relationships with their children require particular attention as positive parenting practices and family relationships can alter predispositions toward substance use later in life, thereby impacting favourably on the cycle of addiction and dysfunctional parenting.

## Background

The human and economic costs of substance use are considerable [1,2]. Although rates of substance use generally are lower for women than for men [3-5], the physical and mental health consequences can be more profound for women [6]. Women who use alcohol and illicit drugs are at particular risk for hepatitis C and HIV infection, and are more likely to have psychiatric co-morbidity and multimorbidity [7]. In addition, substance use during pregnancy and while mothering has negative consequences for children, including risk for prematurity, impaired physical growth and development, physical and mental health problems, and development of substance use problems [8-11].

There is a need for services that effectively and comprehensively address the complex needs of women with substance use issues and their children. In addition to experiencing physical and mental health problems, these women often have personal histories of exposure to physical and sexual abuse and other relationship problems, negative or inadequate social support systems, inadequate income, unemployment, unstable housing, and involvement with the criminal justice system [12-14]. Conners and colleagues [9] suggested that an accumulation of these postnatal environmental risk conditions combined with prenatal substance exposure results in increased childhood vulnerability to poor outcomes. As these authors note, the issues mothers face can "limit their ability to provide for their child's physical and/or emotional needs" (p. 90). Maternal substance use has been associated with limited parenting capacity and an increased likelihood that children are exposed to maltreatment, including neglect [8,15-17], factors that have negative developmental sequelae for children. Children of women with substance use issues are further compromised because they have limited opportunities to develop the social skills and relationships that can help to buffer against risk [9].

Historically there have been separate delivery systems to meet the diverse needs of women with substance use issues and their children. However, there is a growing body of literature reporting on integrated treatment programs that offer a wide range of services (e.g., addictions treatment, parent/parenting counseling, service linkages, and children's programming) in centralized settings for both women and children. These programs have primarily taken two forms: residential and outpatient. Intended treatment length can vary but generally ranges from 12 to 18 months in both types of programs.

Studies that have examined the effectiveness of integrated intervention programs suggest positive outcomes for women and children, including reduced substance use and improved mental health, parenting, and child development outcomes [18,19]. However, the quality of the studies is variable and much of the quantitative research is limited by small sample sizes. This has resulted in inadequate statistical power and an inability to identify moderators of treatment impact.

In a systematic review of 38 studies on substance abuse treatment for women, Ashley, Marsden, and Brady [20] examined specific components of treatment programs and their association with outcomes. Programs with prenatal care or childcare were associated with better outcomes. Orwin, Francisco, and Bernichol [21] conducted a meta-analysis of studies on the effects of substance abuse treatment programs for women on their substance use, maternal well-being, and pregnancy outcomes. Findings suggested that enhancing women-only treatment programs with prenatal care or therapeutic childcare added value above and beyond the effects of standard women-only programs. In recent meta-analyses of the effectiveness of integrated programs for women with substance use issues and their children, we found positive impacts on length of stay, maternal substance use, maternal mental health, and birth outcomes (unpublished data).

While many quantitative studies have examined the effectiveness of integrated treatment programs, there also is a developing body of qualitative and mixed methods literature that encompasses studies conducted to describe the experiences and perceptions of pregnant women and mothers with young children who participate in such programs. As it is important to develop a better-informed understanding of the experiences of participating in integrated treatment programs from women's perspectives, a synthesis of these qualitative data is required.

Meta-analyses of quantitative data and qualitative meta-syntheses share many similar characteristics including: asking of a focused question; establishment of strict inclusion criteria to guide a comprehensive search of the available evidence; and critical appraisal of the located evidence. The two types of reviews are most distinct in the processes for synthesizing findings across included studies, with quantitative meta-analyses utilizing statistical methods to aggregate data and qualitative meta-syntheses characterized by the integration of common findings into narrative themes and the identification of overarching abstract concepts [22]. While quantitative meta-analyses have the power to answer questions about the effectiveness of interventions for specific populations and pre-determined outcomes, qualitative meta-syntheses add to our holistic understanding of issues by providing insight into the processes by which interventions work, factors that facilitate or inhibit the success or uptake of interventions, and the lived experiences of individuals. This paper describes the approach to, and findings of, a qualitative meta-synthesis of findings from women who participated in integrated treatment programs.

The purpose of the qualitative meta-synthesis was to bring individual, high-quality qualitative studies together through a process of comparison, translation, and synthesis of original findings [23]. The specific research questions guiding this meta-synthesis were: 1) What psychosocial processes occur in treatment that contribute to favourable outcomes? and 2) What are the perceived outcomes of integrated intervention programs for women with substance use issues and their children? The research was approved by the Hamilton Health Sciences/McMaster University Faculty of Health Sciences Research Ethics Board.

## Methods

### Search Strategy

A comprehensive and systematic literature search for studies of outcomes and processes associated with integrated intervention programs for women with substance use issues and their children was conducted simultaneously for a quantitative meta-analysis and the qualitative meta-synthesis. The initial search captured literature published up to August 2007. We used three main strategies to identify outcome studies of intervention programs for women with substance abuse issues and their children: online bibliographic database searches; checking printed sources; and requests to researchers [24,25]. First, we searched relevant bibliographic databases (PsycINFO, MedLine, PubMed, Web of Science, EMBASE, Proquest Dissertations, Sociological Abstracts, and CINAHL) for studies published in English, using the terms substance use/abuse, addiction, alcoholism, intervention, treatment, therapeutic, rehabilitation, women, child, mother, infant, mental health, parenting, prenatal, singly and in combination.

Secondly, we examined reference lists of retrieved articles for potentially relevant documents. In addition, we manually searched relevant journals in the area (Journal of Substance Abuse Treatment, Journal of Substance Use, Substance Use and Misuse, Journal of Psychoactive Drugs, Addiction, Journal of Drug Issues, The International Journal of the Addictions, Addictive Behaviors, and the Journal of Substance Abuse). Documents that appeared to be relevant on the basis of titles or abstracts were retrieved.

Finally, we searched for fugitive data (e.g., technical reports, unpublished data). All researchers identified through these searches, as well as researchers presenting at relevant conferences identified using Google and Cross Currents (Upcoming Events), were contacted by email to request any relevant published or unpublished data. Of the 200 researchers identified and emailed, 48% responded and 28 additional studies were identified. In total, 327 studies were retrieved (319 from literature searches and 28 through other forms of searching) and coded for eligibility. A hand-review of all retrieved studies resulted in the identification of 42 papers that included a report of narrative findings from a single qualitative or mixed methods study. The search was updated to capture any research published between the time of the initial search and August 2009, which yielded another three studies with narrative findings.

### Inclusion/Exclusion Criteria

Inclusion and exclusion criteria were developed specifically for the purposes of the meta-analysis and meta-synthesis. Studies had to have explicitly and appropriately defined the study design, the population being served, the intervention and its components, and outcomes or, in the case of qualitative research, processes that contributed to outcomes. Table 1 lists the inclusion criteria used to determine eligibility for the qualitative meta-synthesis. For the purposes of this work, Creswell's [26] definition of qualitative research was used:

**Table 1:** Inclusion Criteria

Study Component	Criteria
1. Study design	• Must explore women's, children's, or clinicians' experiences (outcomes or processes) in an integrated treatment program for substance-using pregnant women or mothers using a qualitative research design that meets the criteria as defined by Creswell [26]

2. Treatment program participants (must meet all criteria)	• Women who are pregnant or parenting
	• Participants had a substance use problem (drug or alcohol) confirmed at baseline enrolment into treatment program by either admission to a substance use treatment program or report of a formal diagnosis

3. Treatment program characteristics (must meet all criteria)	• Must include at least one substance-use treatment service addressing substance use specifically; can be a group or individual treatment service
	• Must include at least one treatment service related to children 0-16 years, including children not yet born such as:
	Prenatal care for the mother
	Childcare or babysitting offered
	Therapeutic childcare
	Child resides with mother in residential treatment program
	Child developmental assessments conducted
	Primary/physical infant health care provided
	Child mental health services or therapy
	Parenting support or education group
	Individual parenting support
	• Treatment program must not include treatment of males
	• Treatment program must not include women who are not pregnant or parenting
	• Treatment program must not be exclusively a smoking cessation program

4. Reported findings	• Qualitative findings addressing processes or outcomes related to any of the following areas:
	Maternal health and well-being
	Child health and well-being
	Parenting

Qualitative research is an inquiry process of understanding based on distinct methodological traditions of inquiry that explore a social or human problem. The researcher builds a complex, holistic picture, analyzes word, reports detailed views of informants, and conducts the study in a natural setting (p. 15).

Two of the authors (WS, SJ) with experience in qualitative research independently reviewed each research report for inclusion in the meta-synthesis. They then met to discuss their individual assessments; when a discrepancy occurred, discussion continued until consensus was met. In the end, 17 of the 45 documents were determined to have met the inclusion criteria. These 17 reports represented 14 distinct qualitative studies, with three reports discussing findings of one study [27-29] and two reports based on another single study [30,31].

### Quality Assessment

Given the lack of a gold standard for assessing the quality of qualitative research [32], we searched for a commonly used rating tool appropriate for our purposes. We chose to use the methodology checklist for qualitative studies developed by the National Institute for Health and Clinical Excellence [33]. The criteria in this tool were adapted from two checklists: criteria for evaluating qualitative studies [34] and 10 questions to help one make sense of qualitative research [35]. This methodology checklist for qualitative studies includes 13 criteria under six broad areas: aims of the research; study design; recruitment and data collection; data analysis; findings/interpretation; and implications of the research. The "Notes on the use of the methodology checklist" provided as an accompaniment to the checklist was consulted throughout the rating process. A summative rating was given based on whether all or most of the criteria were fulfilled (++), some of the criteria were fulfilled (+), or few or no criteria were fulfilled (-). The reviewers agreed that documents that met 10 or more of the 13 criteria would be assigned a ++ rating, those that met 4 to 9 criteria a + rating, and those that met 0 to 3 criteria a - rating. Because guidelines for using the methodology checklist for qualitative studies state that the latter rating implies a study is weak, we decided to exclude studies with this rating from the meta-synthesis.

The two authors who determined inclusion appropriateness also independently reviewed and rated each document for study quality. There was agreement that 7 of the 17 documents met all or most of the criteria (++), 8 met some criteria (+), and 2 met few or no criteria (-). There was disagreement on only one document in that one reviewer rated it ++ and the other reviewer +. As such, 15 reports (representing 12 studies) were deemed to be of adequate quality for inclusion in the meta-synthesis.

### Synthesis Approach

We focused on data that pertained to psychosocial process that contributed to recovery and, secondly, to perceived outcomes for women and their children. Textual data that represented authors' findings and interpretations as well as verbatim data from study participants were extracted. These data were copied into Word documents, which were then imported into QSR International's NVivo7 program.

Thematic analysis of data was conducted using the approach suggested by Atkins et al. [36]. We first arranged the documents in chronological order, starting with the oldest. This allowed the meta-synthesis to capture developments in knowledge related to integrated programs for women with substance use issues and their children over the 14-year span of the studies. We then created a preliminary grid to display themes and concepts within each study under broad headings that reflected the purpose of the meta-synthesis: processes and outcomes.

The lead author independently analyzed the data from all documents while the second author analyzed the data from half the documents, specifically, alternate documents from the chronological list. Themes became increasingly refined through reciprocal translation, that is, the translation of studies into one another by comparing the themes and concepts in one account with those in others [36]. The conceptualization of ideas was further refined as the analysis proceeded using an iterative approach. That is, as new ideas emerged, articles that previously had been analyzed were reviewed a second time to look for instances of these ideas and to ensure consistency in the approach to coding.

The two lead authors met to discuss their findings part way through the analysis to discuss themes arising from the reciprocal translation. Much of the discussion focused on comparing and contrasting the more abstract analytical themes related to processes. After another period of independent coding of remaining documents, these authors met a second time to reach consensus on themes, which had become more refined and interpretive in nature. A higher order or synthesized translation was achieved.

## Results

### Characteristics of Included Studies

The characteristics of the studies included in the meta-synthesis are presented in Table 2. Two of the reports were masters dissertations [37,38], two were doctoral dissertations [27,39], and one [18] was a Special Supplement published in the Journal of FAS International. The other documents were journal articles, with two of these reporting on Nardi's dissertation research [28,29]. All of the studies were conducted in North America (eight in the United States and four in Canada). Most used a qualitative descriptive design and collected data using semi-structured face-to-face interviews. Six studies gathered data in whole or in part from women who had completed an integrated treatment program [37-42], and thus were positioned to report on outcomes perceived to be attributable to program participation.

**Table 2:** Study Characteristics

Author(s)	Setting/Program Elements	Objective	Research Method	Study Participants	Qualitative Data Source(s)
Nardi [27,28]	Midwestern city, USA An intensive outpatient perinatal addiction treatment program for pregnant and parenting women and their children newborn to 3 years of age Services included: a therapeutic nursery; detoxification program; medical services; 12-step program and other addiction education programs; outpatient services (transportation, child care and meal support); chemical dependency treatment, parenting training program; counseling and psychotherapy; and skills training	To explore the nature of parenting and addiction recovery for pregnant and parenting women in an addiction treatment program	Mixed methods combining grounded theory methodology with quantitative methods of descriptive and differential statistics	N = 17 Low-income, single women 20-37 years old, with a mean age of 28 years Most (82%) were African American, lived in the inner city, and were involved with child protection services	Single semi-structured interviews, participant observation, field notes, client records (medical records, infant birth records, therapy treatment notes, program progress notes)

Nardi [29]	As above	To explore the nature of parent-infant interaction during the first year in a perinatal addiction treatment program	As above	As above	As above

Baldwin et al. [44]	Western USA *Mom Empowerment, Too! (ME2) Program*, a community-based intervention with multiple program modalities delivered by pubic health nurses including: home visits; case management; resource referrals; and a series of 16 educational-support sessions focused on substance use, pregnancy, nutrition, self-nurturance, responsible parenting, development of life skills, problem solving, and stress management Children participated in a program focused on child health and development	To examine women's experiences in a community-based program for young mothers (and their children ages birth to 5 years) involved in substance abuse and their perceptions of risk and health promoting behaviours before and during the intervention program	Qualitative description using ethnographic interview techniques within a participatory action research process	N = 42 Low-income, pregnant women and mothers 18 to 33 years old Most (83%) were European American, 14% were Hispanic, and 0.02% were African American	Semi-structured interviews at each of the 16 program sessions

Howell & Chasnoff [46]	Eastern USA Evaluation of five *Improve Care for Pregnant Substance Abusers *demonstration sites funded by the Health Care Financing Administration in Maryland, Massachusetts, New York, South Carolina, and Washington These state-developed programs provided services to improve access to care for pregnant substance abusers by providing enhanced services and coordinated prenatal and substance abuse care	To identify factors in women's lives that facilitate or act as barriers to the treatment process and to describe successful program components that addressed the needs of the population	Qualitative description	Three types of participants: 1. Program administrators (n = 25) 2. Care providers (n = 147) 3. Pregnant and postpartum women (n = 88) Program providers included registered nurses, physicians, case managers, outreach workers, and therapists	Thirty-three focus groups were conducted across the five sites including: 5 groups of program administrators; 16 groups of providers; and 12 groups of women participating in the programs

Schretzman [39]	New York City, USA *Casa Rita *residential program for homeless pregnant women and mothers with addiction problems and their children Program components included: individual, group and family therapy; on-site child care; and private residential accommodation	To identify factors associated with successful treatment outcomes and to identify factors that both support and challenge participants' post-treatment experiences	Mixed methods with a qualitative case study conducted concurrently with a descriptive quantitative study	N= 20 women who had completed the program and remained alcohol and drug free at the time of the study	Single in-depth, semi-structured interviews

Salmon et al. [43]	San Jose, California, USA An intensive, 9-month outpatient drug treatment program for pregnant women and parenting substance abusing women The program was based on a 'one-stop shopping model' and on-site services included: child care; transportation to and from the program; individual and group counseling; a 12-step recovery program; education on a variety of health and social issues; parenting skills; development of life skills; referrals to community services; and intensive case management	To explore the perceptions of pregnant and parenting substance-abusing women in an outpatient drug rehabilitation program about provider and social support, and to identify program elements that supported maintaining their abstinence from substance use	Qualitative description	N = 20 Average age was 30 years; 55% of participants were Hispanic and 20% were Caucasian; average number of children was 3.3 The majority were single (70%) and unemployed with public assistance (95%)	Two semi-structured questionnaires with open-ended questions and structured questions on demographics and drug history completed during a private interview

Kunkel [37]	Abbotsford, British Columbia, Canada A residential treatment facility for addicted women and their children offering a 10-week program Treatment services included: daily counseling and psycho-educational groups; individual counseling; parenting training; an exercise program; and support meetings On-site licensed daycare was provided	To understand mothers' lived experiences of participating in a residential treatment program with their children, and to study the impact of the involvement of children in their mothers' residential addiction treatment program on both the experience of treatment and on recovery	Phenomenology	N = 6 Age range 21-36 years; five women were Caucasian and one was Aboriginal	In-depth, open-ended interviews during week 8 of the program and a follow-up interview 1 month following treatment discharge

Simpson [38]	Windsor, Ontario, Canada A community-based harm reduction treatment model that offered outpatient services for chemically dependent pregnant women and parenting mothers The 17-week program offered: addictions, parenting, children's, and health programming; support for accessing transportation, housing, and food; and a parenting program delivered one afternoon a week	To explore women's life situations and perspectives of the impact of the parenting program on their parenting style and relationship with children	Mixed methods, predominantly qualitative description informed by case study and phenomenological approaches	N = 7 who completed the 17-week parenting program module; most continued to attend the program for support Average age of study participants was 35 years; six mothers were Caucasian and one mother was Aboriginal	Single semi-structured interviews 3 months after program completion

Sword et al. [42]	Hamilton, Ontario, Canada *New Choices*, a comprehensive community-based "one stop" program of service delivery for women with substance use issues who are pregnant or parenting young children Program components included: addiction groups and counseling; nutrition counseling and skill development; parenting education; peer support; and an enriched children's program Linkages with prenatal services, a physician, and a perinatal home visitation program also were available	To describe mothers' experiences of participating in the community-based treatment program and to understand their perceptions of how the program influenced changes in their lives and the lives of their children	Qualitative exploratory	N = 11 women ages 21 to 36 years who had completed at least 3 months of the program	Seven women new to the program completed an in-depth individual interview and seven women participated in a single focus group post program involvement Two of the seven women completed a follow-up interview at 3, 6, and 12 months post program involvement, four women completed two follow-up interviews, and one women completed one follow-up interview

Motz et al. [18]	Toronto, Ontario, Canada *Breaking the Cycle*, a community-based early identification and prevention program for pregnant women and mothers who are using alcohol or other substances, and their young children The program provided mothers with a single point of access to a range of multi-sectoral, integrated services: individual and group addiction treatment; parenting programs; child care; child development services; health/medical services; Fetal Alcohol Spectrum Disorder diagnostic clinic; mental health counseling; case management; parent-infant counseling; home visitation; pregnancy outreach; and instrumental support	To explore factors influencing women's progress through and satisfaction with the treatment program services	Program evaluation using mixed methods, including a qualitative descriptive component	N = 19 Demographics specific to the women who participated in the focus groups were not provided	Three separate focus groups: 1. Women participating in the pregnancy outreach program (n = 7) 2. Women recently transferred to ongoing/active service (n = 5) 3. Women receiving ongoing/active service for more than 12 months

de Guzman et al. [40]	New York City, USA *Family First Intervention*, a multi-session, individually-based behavioural intervention program for mothers with patterns of problem drinking who infected with or at-risk for HIV The intervention consisted of 14 sessions; the first seven sessions supported mothers in reducing or eliminating problem drinking and/or drug use and the final seven sessions focused on the development of skills for parenting adolescents	To examine program participants' experiences in the program and to describe their perceptions of intervention processes that influenced behavioural changes related to substance use, parenting behaviours, coping, and social support networks	Qualitative exploratory	N = 25 selected from a larger intervention trial The full sample comprised women of colour, with 64% African American, 32% Latina and 4% multiracial; average age was 41 years All were receiving Medicaid and 60% were HIV infected	Single in-depth semi-structured interviews after completion of the final quantitative follow-up (12 to 20 months after the last intervention session)

Polansky et al. [41]	Philadelphia, USA A publicly funded residential treatment program for women with addictions and their children Program elements included: weekly individual psychotherapy; family therapy; a 12-step group program for treating addiction; a trauma group; a healthy relationships group; a parenting group with an emphasis on psycho-education; and an optional 6-week attachment-based parenting group	To explore mothers' experiences of participating in the attachment-based parenting group and their perceptions of how the group influenced interactions with their children and children's behaviour	Qualitative exploratory	N = 7 All, with one exception, were African American; six women were in their 20 s or 30 s and one woman was in her 40 s	Single semi-structured interviews 1 to 3 weeks following completion of the parenting group

Wong [31]	New York City, USA Participants were recruited from four residential programs providing addiction treatment services to mothers and their children All of the programs offered both substance abuse treatment and parenting programming	To explore mothers' perceptions of the supportive function of the treatment program and how it affected their parenting experiences and outcomes	Mixed methods, predominately qualitative exploratory with a descriptive quantitative component	N = 10 women 25 to 45 years of age who had completed at least 3 months of treatment	Three in-depth, semi-structured interviews, participatory observation, and field notes

Wong [30]	As above	To explore how substance-abusing mothers perceived their parenting experiences within the social context of a residential treatment program	As above	As above	As above

### Processes

Women experienced a number of psychosocial processes during treatment that played a role in their recovery and contributed to favourable outcomes. These processes included: development of a sense of self; development of personal agency; giving and receiving of social support; engagement with program staff; self-disclosure of challenges, feelings and past experiences; recognizing patterns of destructive behaviour; and goal setting. A final process, the motivating presence of children, sustained women in their journey to recovery. The sources of these process themes are shown in Table 3.

**Table 3:** Summary of Sources for Process Themes and Outcomes

Processes	Sources
Development of a sense of self	Baldwin et al. [44]; de Guzman et al. [40]; Kunkel [37]; Motz et al., [18]; Nardi [27-29]; Salmon et al. [43]; Schretzman [39]; Sword et al. [42]; Wong [30,31]

Development of personal agency	Baldwin et al. [44]; de Guzman et al. [40]; Kunkel [37]; Nardi [28,29]; Simpson [38]; Sword et al. [42]; Wong [30,31]

Giving and receiving of social support	Baldwin et al. [44]; Howell hasnoff [46]; Kunkel [37]; Motz et al. [18]; Nardi [27-29]; Polanksy et al. [41]; Salmon et al. [43]; Schretzman [39]; Simpson [38]; Sword et al. [42]; Wong [30,31]

Engagement with program staff	de Guzman et al. [40]; Howell hasnoff [46]; Motz et al. [18]; Salmon et al. [43]; Schretzman [39]; Simpson [38]; Sword et al. [42]; Wong [30,31]

Self-disclosure	Baldwin et al. [44]; de Guzman et al. [40]; Kunkel [37]; Nardi [27-29]; Polansky et al. [41]; Schretzman [39]; Wong [31]

Recognizing destructiveness patterns	Baldwin et al. [44]; de Guzman et al. [40]; Howell hasnoff [46]; Nardi [27-29]; Kunkel [37]; Salmon et al. [43]; Schretzman [39]; Simpson [38]; Sword et al. [42]; Wong [30]

Goal setting	Baldwin et al. [44]; de Guzman et al. [40]; Kunkel [37]; Simpson [38]; Sword et al. [42]; Wong [31]

Motivating presence of children	Kunkel [37]; Schretzman [39]; Simpson [38] Sword et al. [42]; Wong [30,31]

**Outcomes**	

Maternal outcomes	de Guzman et al. [40]; Kunkel [37]; Schretzman [39]; Simpson [38]; Sword et al. [42]

Child outcomes	Sword et al. [42]

Parenting outcomes	de Guzman et al. [40]; Kunkel [37]; Polanksy et al [41]; Simpson [38]; Sword et al. [42]

#### Development of a Sense of Self

One commonly identified process that emerged as part of addiction recovery and was first reported by Nardi [27-29] was *development of a sense of self*. This included development of a sense of self-worth, self-identity, and self as a partner in a relationship.

Nardi's [27-29] research revealed an increasing sense of self-worth during program involvement. As she noted [27], women "began to see themselves as persons who were changing and who deserved help" (p. 138). Women in this study also began to recognize they had strengths and needed to build on these strengths to improve their lives. Salmon, Joseph, Saylor, and Mann [43] commented on women's developing self-worth in relation to being "a better person when off drugs" (p. 243). Similarly, Kunkel [37] described how women's sense of personal worth developed in parallel with the desire for recovery and realization that they "don't even need drugs" (p. 79). While Kunkel found that women began to see themselves as having value independent of their children, women also described having value because they had children and because they were valuable to their children. Consistent with this notion of self-worth as a mother was the finding of another study that women started to value not just themselves but their parental selves in particular [31].

As women moved through the recovery process, Nardi [27] remarked that they began to form "an identity as a coherent, separate self" (p. 139). Wong [31] likewise commented on the development of self-identity in that women showed "an emerging ability to separate their own needs from those of others" (p. 127). At the same time, she noted that women developed an ability to integrate different aspects of self, including self as an addict, mother, woman, and daughter. As one woman in this study said, "If I didn't admit that I did have a drug problem then I wouldn't be a mother to my son" (p. 128) [31].

Another transformation that impacted self-identity was that women developed greater awareness of their children and their maternal roles [27-29]. Wong [31] remarked on women's developing maternal empathy and the ability to more easily identify with children's needs and emotions. As a result of an enhanced maternal identity, women not only became more conscious of responding to their children's needs, but also were able to bond with their children and began to view them more positively [27-29,31]. Through the development of a positive parental self-concept women became motivated to learn parenting skills and overcome psychological barriers to parenting [31].

Nardi [27-29] noted that although it was important for women to be part of a group that did not require overly intimate relationships, the presence of other women and children enabled them to connect with others and to build relationships. They began to see themselves as partners in a parent-child relationship [27-29]. Ultimately, women experienced improved relationships with their children and an enhanced parental self-concept and parenting [31]. Women in Schreztman's [39] study reported that relationships with their children were important to staying sober.

Women also developed a capacity for healthy relationships and a sense of self as a partner in relationships with friends, partners, family members, program staff, and other program participants [27-30]. Baldwin, Rawlings, Marshall, Conger and Abbott [44] commented that women discovered "the importance of developing trusting relationships and positive friendships" (p. 381), which women noted required that they first trust themselves. In addition to an ability to trust others, some studies revealed other factors that may be important in developing capacity for relationships. For instance, de Guzman and colleagues [40] found that women developed the ability to identify and express their needs to others while Wong [31] reported that women developed the capacity to form partnerships with others to pursue mutual goals.

Group interaction and discussion were instrumental to self-development. Interactions with other mothers facilitated maternal self-awareness through role modeling, discussion, and positive feedback [27-29]. Group discussion also fostered self-examination of lives and choices and, ultimately, self-discovery in a safe environment [27-31,42]. Additionally, the encouragement of group members was important to the building of self-esteem and maintaining faith in one's ability to be successful in achieving goals [18,42].

#### Development of Personal Agency

Women experienced *development of personal agency *during program involvement. As defined by Smith and colleagues [45], personal agency is the capacity to achieve desired outcomes on one's own behalf through ability, choices, perseverance or planning. Women overcame powerlessness [30,31] and began to discover "their own agency, power and growth" (p. 381) [44]. Recognition of strengths and having a sense of control contributed to improved self-esteem, self-worth, and confidence [31,42,44].

Development of personal agency fostered capacity for change. Women developed a willingness to accept personal responsibility for change, recognizing that they were the only ones who could regain control over their lives [37,42,44]. Women commented specifically on their capacity to decrease substance use and resist the urge to relapse, and on their confidence in being able to overcome their weaknesses and stay sober [37,42]. An important aspect of change in substance use was the development of alternative coping skills, such as relaxation techniques, to replace substance use as a coping mechanism [28,29,40,42]. Recognizing cues to relapse enabled women to plan in advance for confronting risks through the use of substitute coping responses [28,29,38].

#### Giving and Receiving of Social Support

Many of the studies highlighted the *giving and receiving of social support *as being instrumental to women's recovery. Nardi [28] described this support as being "embedded in the interpersonal interactions that took place at the program, and occurred in a feedback loop of give-and-take among women" (p. 85). Others similarly described the interactional nature of social support that occurred within treatment groups [18,23,37,38,43,44]. Wong [30] commented that past experiences created ambivalence about seeking support, such that women had to learn "to trust the support at their own pace" (p. 167). The nature of the social support received within program groups might ultimately have enabled women to accept help without resentment, obligation, and pressure that can cause additional stress [27-29]. Moreover, some studies reported that the support often served to lessen or buffer women's multiple stressors [30,31,43].

Through interaction, women were afforded the opportunity to understand and work through their problems while being provided support and encouragement [30,31,46]. Positive relational experiences instilled confidence in their ability to be successful in the recovery process and enhanced perceptions of self and others [30,42]. Women also gave and received feedback and advice to one another [38,43]. In some instances, they learned from each other through role modeling of parental behaviour and sharing experiences [18,27,29]. Women ultimately felt that others respected and cared for them [38,39]. The ability to mobilize the support of others suggests that in addition to developing personal agency, women also developed interpersonal agency [45].

The importance of social support being provided by others with a shared past is noteworthy. Within the group programs women felt safe and were able to talk with others who had similar experiences without being judged or manipulated [18,27-29,31,38,39,42,43]. In turn, they came to trust others [37,38,44]. Women were comfortable sharing their past and being open about their experiences because they felt understood and could provide understanding to others [18,38,41,42].

Listening to the stories of women who were improving their lives gave women encouragement and a sense of hope [42]. Motivation for recovery also was prompted by hearing stories of women who had lost custody of their children [18,37]. Finally, the relationships with other women in similar situations decreased feelings of isolation and disconnection and, in some instances, genuine friendships developed [18,31,38,42].

#### Engagement with Program Staff

*Engagement with staff *emerged as a process that was central to women's participation in the programs and behaviour change. The non-judgmental approach of staff and attributes such as compassion, honesty, empathy, and respect facilitated the development of therapeutic relationships [18,31,38-40,42]. These characteristics often were perceived to create a caring, safe, and supportive environment for recovery [31,39,43]. Additionally, feeling understood "as a whole person, and not just as a substance user" was important to women (p. 53) [18].

A non-directing approach by staff was important. Women valued being assisted to understand their problems and what contributed to them, and to identify strategies to address them [43,46]. They also appreciated being able to set their own agendas at their own pace, being provided treatment options, and being supported in their choices and decisions [18,42]. The ability of staff to listen also was significant to women in that it not only promoted understanding and facilitated problem solving, but also conveyed respect [18,31,39,43].

Motz and colleagues [18] and Wong [30] commented that women's relationships within the treatment facility are transformative because they are growth-promoting and empowering. In contrast to previous, often complex and challenging relationships, the ones with staff are can be negotiated and are characterized by a sense of connectedness, openness, caring, and respect [18,31,40]. Wong reported that the characteristics of mother-staff interactions, such as the offering of empathy, were often paralleled by women in their interactions with their children [30].

Two studies reported on children's engagement with staff. Sword and colleagues [42] noted that women commented favourably on the staff's interaction with children and that children felt comfortable in the program while the report by Motz and colleagues [18] included a mother's remark about her daughter's closeness to a therapist and how she looked forward to seeing her. Valuing children's relationships with staff may be attributed, in part, to women's developing awareness of children's needs.

#### Self-disclosure

The nature of the relationship with group participants and staff facilitated *self-disclosure*, which was an important component of the recovery process [39]. As reported by de Guzman and colleagues [40], "women freely shared the challenges they faced with alcohol and drug use, free of judgment" (p. 1259). Schretzman [39] and Polansky and colleagues [41] similarly commented that women were able to open up, and felt comfortable sharing their feelings and their past in a safe environment. As such, they were able to be honest with themselves and others [31,38].

Through self-disclosure, women were able to confront the past. Often this revolved around women's relationships with their mothers and the painful experiences of abandonment, neglect, and abuse [27-29,31,39,41,44]. Awareness of the negative impact of such relationships provided insight into parenting and fostered a more positive approach to parenting their own children [37,41].

#### Recognizing Destructiveness Patterns

Another process fundamental to change and recovery was *recognizing destructiveness patterns*. Women were enabled to examine their lives and choices [27-29], and often began to recognize the extent of their substance use problem only after encountering women with similar difficulties and hearing their stories [37,42]. They developed awareness not only of the impact of their drug or alcohol use on themselves and their body, but also the impact of other choices such as staying in abusive relationships and continuing contact with others with substance use issues [27-29,43,44]. Moreover, women became cognizant of the effects of their choices on their children [27-29,38,40]. While acknowledgement of the effect of substance use on their children promoted women's continued participation in program activities, they also had to overcome associated feelings of guilt and anxiety [30,31,38].

Given the negative influence of others who use substances, it was important that women had the opportunity to develop and maintain relationships with sober peers [39]. Such individuals served as motivating role models and sources of emotional support [39,46]. Women's connectedness with sober peers contributed to feeling cared for and assisted women in maintaining sobriety [39].

#### Goal Setting

An activity undertaken with the support of program staff that contributed to treatment success was *goal setting*. It was important that women's goals were realistic, and staff played a role in determining the appropriateness of goals in relation to the program's resources, time frame, and purpose as well as in relation to women's specific needs [40]. Goal setting was motivating for women and provided a means to prove to themselves that they could accomplish something meaningful [44]. Both the setting of and achieving of goals gave women a sense of pride and accomplishment [40]. Reviewing women's goals and progress towards them at the outset of each program session allowed for goals to be revised as women's needs and circumstance changed [40].

While personal goals were variable, women often entered treatment wanting to become better parents and to establish their parental role [30,31,37,38]. An important goal for many women was maintaining or regaining custody of their children [38,42]. In situations where children were under the protection of child welfare, women were motivated by the possibility of their children being returned to them [38].

#### Motivating Presence of Children

A major finding of Schretzman's study was the importance of having children present during treatment in that it helped sustain women's motivation in their recovery process [39]. Other research similarly noted the value of children's presence as a motivating influence [30,31,37,38,42], which is not surprising given the centrality of motherhood to women's identity and recovery. As Kunkel [37] commented, "Children's presence in treatment provides women with security and comfort in their role as parents and in their recovery" (p. 69). Wong also commented on the emotional support afforded women simply by having children in program [31]. In addition, their presence often contributed to an awareness of the urgency of dealing with recovery issues [39].

Women reflected on the value of learning to parent with their children by having them in the program [31,37,38]. Children's presence afforded a base for mothers to build on their parenting experience and to improve parenting skills through experimentation [31]. The availability of children also made it possible for women to re-establish relationships with them and to deal with the guilt associated with their past parenting behaviour by learning better parenting practices [31,37-39]. In some instances, the program provided an opportunity for supervised visitation with children, which was emotionally satisfying to women [31,42].

### Outcomes

Perceived outcomes of participating in an integrated intervention program included: a maintained sense of personal agency; improvements in personal well-being; sustained sobriety or decreased substance use; establishment of positive social support networks; greater insight into self, others, and relationships; increased access to community services; and a more positive approach to professional relationships. Women also perceived that their capacity for parenting was enhanced in that they reported increased knowledge, skills, and confidence. They also identified improved maternal-child communication and relationships. Studies included in this meta-analysis did not explore women's perceptions of child health outcomes, with the exception of the study by Sword and colleagues in which women reported that program involvement had a positive impact on their children's behaviour and development [42]. The sources of specific outcome findings are shown in Table 3.

#### Maternal Outcomes

A sense of personal agency reportedly was maintained post-treatment. Women commented on their increased sense of control and confidence [42], and how they exercised personal power in making careful choices about with whom they associated and in enforcing boundaries in relationships [37]. As reported by Kunkel [37], they also demonstrated a "pro-active" approach to life that enabled them to continue with recovery activities and life goals (p. 61). Women were able to sustain sobriety or their decreased substance use, often with the assistance of an existing social network of sober friends or new support networks of substance-free associations [38,39,42], and exercised the self-discipline required for successful recovery [37].

Women experienced improved personal well-being and a readjustment of priorities in that they were more content with self and life, and had learned to relax and enjoy life and their successes [37]. Sword and colleagues [42] commented on women's employment readiness, which enabled them to achieve goals related to obtaining one's own pay cheque. In addition, women had greater insight into their strengths and weakness, and increased awareness of people and relationships [37]. It was noted that women had enhanced access to other services to benefit themselves and their children post-program [40,42]. Kunkel [37] importantly observed that women had a more positive approach to professional relationships and entered them with a "spirit of cooperation" (p. 61).

#### Child Outcomes

The study by Sword and colleagues [42] was the only one to comment on child outcomes. Women observed that program involvement had a positive impact on children's behaviour and development, including their motor, social, and language skills.

#### Parenting Outcomes

A number of studies reported enhanced capacity for parenting as an outcome of program involvement. Women gained increased knowledge about parenting and learned new parenting skills, which they willingly applied at home with their children [38,42]. They became knowledgeable about specific strategies, such as the use of rewards, and how to use positive discipline techniques [38,42]. Women also learned the importance of verbal communication [38,40,41]. In particular, as reported by Polansky and colleagues [41], they came to appreciate the use of communication as an alternative to physical punishment, and its value in teaching and guiding children and in strengthening emotional bonds. In addition, women gained awareness of the importance of listening to their children [40,41].

Through involvement in treatment programs, women developed increased understanding of children, their behaviour, and their needs [38,40,42]. Simpson [38] commented specifically on women's awareness of children's emotional needs. The enhanced understanding of children strengthened maternal relationships with them [42]. Women also described more positive engagement with their children [37,38,40]. This included involvement in day-to-day activities at home, such as having dinner or watching a movie together, and attending children's games as well as finding services to help children with their issues [38,40].

Finally, women developed increased confidence in parenting and a positive identification with their parenting role [38]. As one woman in Simpson's [38] study said, "I can see how my perspective has changed in that I don't have to be a perfect mother in order to be a good mother...that every day doesn't have to be perfect" (p. 94).

## Discussion

Many distinct but interconnected processes emerged as being important to women's addiction recovery. For the purposes of this discussion and consistent with the findings of the meta-synthesis, recovery is not simply a process of reducing use of drugs and alcohol or attaining abstinence from drugs and alcohol. Rather, recovery is regarded as a process of "re-covering" oneself, that is, as a process of personal and individualized growth that unfolds along a continuum, leading to a higher quality of life [47,48].

Through involvement in programs, women developed an enhanced sense of self. Development of a sense of self-worth is notable as women with substance use issues tend to have low levels of self-esteem [49] and pregnant women and mothers, in particular, experience stigma that negatively impacts their self-worth [50,51]. Along with an improved self-worth, the development of a non-addict identity is an important aspect of recovery [52,53]. Mothers with substance use issues often experience difficulties in developing a maternal identity, which is related to limited care giving experience, a focus on one's own needs for recovery, and addressing only the non-emotional needs of children [49]. The meta-synthesis revealed that integrated treatment programs support women in defining self in other roles, including self as mother.

As part of the recovery process, and related to the development of a non-addict identify, women also developed a capacity for relationships with their children and with others internal and external to the treatment program. This is an important aspect of recovery because women who use substances often have histories of physical, sexual or emotional abuse as children [54,55]. These histories, along with socioeconomic circumstances and characteristics of drug-exposed children, can negatively affect parenting practices [56]. In addition, women with substance use issues often continue to experience abuse beyond childhood [55], which may negatively impact their abilities to engage in adult relationships.

Another finding of the meta-sythesis was that women developed personal agency through integrated treatment program involvement. Other first person accounts similarly describe people in recovery as active agents of change in their lives rather than passive recipients of services [48]. This cognitive resource plays an important role in promoting healthy behaviours, including continued abstinence [57]. As reflected in the findings of the meta-synthesis, an aspect of developing personal agency in recovery is learning and being able to implement adaptive coping strategies as alternatives to substance to cope with stress [47]. The lives of women with substance use issues tend to be characterized by chronic life stress related to issues such as abuse and traumatization, single parenting, inadequate income, family or social problems, health problems, and removal of children from the home [49,58]. Because stress can trigger relapse, adaptive coping strategies are key resources that promote maintenance of recovery [47,59].

The finding that women experienced the giving and receiving of social support within integrated treatment programs is noteworthy as women who use substances often experience social isolation and limited social support [49,60]. Social support, including that of peers and helping professionals, is a key resource in recovery [47,48,61,62]. As suggested by the meta-synthesis findings, social support may act to buffer against stress, a notion espoused by Tucker and colleagues [63] who found that women with greater social support reported less substance use. It is essential that women have social support systems that are constructive and provide support without enabling drug use [64]. Moreover, as noted by Kellogg and Kreek [52], a constructive social setting enables women to develop relational or interpersonal aspects of their self-identity, a component of self-development that emerged in the meta-synthesis.

Getting women to engage and participate in recovery activities is a first step to treatment retention and longer-term outcomes [38,65]. Because staff members are key actors in this process [65], the finding that women engaged with program staff is important. Therapeutic relationships were characterized by acceptance, a non-judgmental attitude, openness, empathy, and respect. In a recent study, stronger therapeutic engagement, specifically program participation and rapport with staff, was found to be associated with higher treatment motivation and readiness and to better psychosocial functioning (e.g., higher self-esteem, self-efficacy, decision making, social consciousness) [66].

Therapeutic engagement with staff and the support of women with shared experiences facilitated self-disclosure of histories of neglect and abuse and substance use-related issues. In a study of women in recovery, "breaking the silence" by discussing abuse experiences assisted them in reshaping their sense of self and connecting to others [67]. Altering one's concept of self and committing to a newly established sense of identify was seen as integral to the process of recovery. Women in this study also reported that they began to understand their behaviour patterns, which enabled them to take action.

Recognizing destructiveness patterns was a theme revealed by the meta-synthesis. It is consistent with the concept of "truthful self-nurturing" that was identified by Kearney [68] as the basic process of women's addiction recovery in a review of 10 study reports of recovery processes. As she noted, women developed insight into the consequences of substance use, including harms to their children, and a gradual realization that alcohol and drug use caused more distress than it relieved. "Giving in to the hard truths" enabled women to engage in more healthful ways of caring for themselves and to develop positive relationships (p. 503) [68]. They became aware of the need for behaviour change, and while often women initially focused on simple goals such as eating and obtaining shelter, they progressed to more complex long-term goals [68].

Goal setting was another theme that emerged from the meta-synthesis. The ability to engage in future-oriented, goal-directed behaviour is central to the recovery process in substance use treatment [52]. Programs that are supportive and goal-directed demonstrate improved treatment participation and better outcomes [52,69]. The need for women to act in a consistent self-directed manner to achieve goals underscores the importance of personal agency [52].

The qualitative meta-synthesis highlighted the motivating presence of children for women to remain in integrated treatment programs. The meta-analysis of quantitative studies of integrated treatment programs we conducted (unpublished data) revealed that, relative to non-integrated programs, having children present increases client engagement, possibly because pregnancy-, parenting-, and child-related services increase maternal motivation to actively engage and remain in treatment [42]. Creamer and McMurtrie [70] commented that women in treatment often have difficulty or are unwilling to separate themselves from their role as mother to focus exclusively on their own needs. They, too, identified that children can be motivators for staying in treatment. Moreover, because mothering can build self-esteem [70], the presence of children also contributes to women's development of sense of self.

The findings regarding outcomes are consistent with previous reviews and meta-analyses of quantitative studies in supporting the effectiveness of integrated programs in improving maternal engagement, substance use, and well-being. This qualitative meta-synthesis also identified positive outcomes of integrated programs for parenting knowledge, skills, and confidence, as well as child development and behaviour and the parent-child relationship. The lack of attention to child outcomes is an important omission of the qualitative studies conducted to date, especially given that integrated programs are designed to meet the needs of women and their children.

Strengths of this meta-synthesis include the use of: a systematic and comprehensive search strategy; criteria that were congruent with the defined purposes of the synthesis to make inclusion decisions; and a quality assessment checklist developed by the National Institute for Health and Clinical Excellence (NICE) [33]. Several of the studies were determined to be of high quality, and lower rankings tended to represent an absence of information (e.g. research methods unclear, study limitations not reported, inadequate discussion of study limitations) rather than explicit methodological inadequacies. Details about themes and verbatim quotes were extracted from the documents. These data were then synthesized using a systematic and iterative process to create high-order constructs or themes that reflected women's experiences. Finally, two of the authors independently determined whether reports met inclusion criteria, assessed quality, and analyzed the extracted data, with decisions made by consensus when there were differences. While we anticipated that conducting a thematic analysis of the studies in chronological order would capture developments in knowledge, we found no discernable developmental patterns. This most likely is related to the wide diversity in study aims and research methods.

The main limitation of this meta-synthesis is that it relied on data derived predominantly from simple qualitative descriptive studies. Within the hierarchy of qualitative evidence [71], descriptive studies are a weaker type of evidence compared to conceptual studies or generalizable qualitative studies conducted using more theoretical approaches such as grounded theory or phenomenology. Descriptive studies can confirm and describe characteristics of a phenomenon within a specific population and highlight context-specific issues, but may have limited transferability to contexts outside of the original study and limited utility for evidence-informed practice and policy development. While it has been argued that it is inappropriate to synthesize the findings of qualitative studies that have been carried out in diverse contexts and to generalize across studies, qualitative meta-syntheses can produce new understandings through conceptual development of shared meanings or generative mechanisms [22,72].

## Conclusion

The process findings of the meta-synthesis support the need for programs to adopt "wellness-oriented practices" that improve personal health and social functioning [47,73]. Given the importance of a positive sense of self, inner strength, and social support in fostering these outcomes [73], the notion of a "healing environment" characterized by empowerment, safety, and connections becomes salient [50]. Covington [50] suggested that connections with treatment staff that are empathic, respectful, and compassionate contribute to the development of connections among program participants through modeling of similar behaviours. Such growth-promoting relationships with helping professionals and peers have been identified as having a key role in women's recovery and highlight the need to adopt a relational model of treatment [50,51,70].

Relational models of treatment take into account past and current family relationships, relationships with friends and partners, relationships with children, and relationships developed within the treatment context [50,51]. Given women with substance use issues often have experienced multiple "disconnections" in their lives, including neglectful or abusive parenting, failed relationships, and experiences of violence in adulthood, it is important that they are assisted in developing healthy relationships with others [51,62]. In addition to building relational skills, interactions within the group can contribute to the development of a non-addict identity [52] and also support transformative learning, which is an effective model for the recovery process [74].

The phases of transformative learning are congruent with many of the process findings of the qualitative meta-synthesis. An initial step of transformative learning is self-reflection during which an individual tries to make meaning of one's life and experiences [74]. Involvement with others is essential for learning to continue as "rational discourse" provides opportunity to listen to the opinions and experiences of others, prompting recognition of a shared process of transformation and exploration of options [74]. Acquisition of knowledge and skill to implement plans, planning of a course of action, and trying out new roles are other integral components that lead to action and ultimately maintenance and reintegration phases [74]. Because transformational learning can be painful [74], it is important that it occur in the context of a safe, supportive environment.

Women's relationships with their children need particular attention. Programs should provide support for the parenting role through a number of strategies, including: providing assistance in gaining insight into personal experiences with dysfunctional parenting and their influences on current parenting [49]; education on child behaviour and development [49]; opportunity to discuss the effect of substance use on children [49]; and practice of communication and discipline techniques [75]. As suggested by this meta-synthesis, having children present in the treatment program is highly motivating, and facilitates the development of parenting skills and the reestablishment of relationships with children. Positive parenting practices and enhanced family relationships influence healthy child outcomes and alter predispositions toward negative behaviours, thereby playing a significant role in breaking the cycle of addiction and dysfunctional parenting [49,75,76].

## Competing interests

The authors declare that they have no competing interests.

## Authors' contributions

All authors contributed to the original study design. WS and SJ conducted the relevance and quality assessment, extracted data, carried out the thematic analysis, and prepared the draft of the manuscript. All authors read and approved the final manuscript.
